# Age Related Functional Connectivity Signature Extraction Using Energy-Based Machine Learning Techniques

**DOI:** 10.3390/s23031603

**Published:** 2023-02-01

**Authors:** Sravani Varanasi, Roopan Tuli, Fei Han, Rong Chen, Fow-Sen Choa

**Affiliations:** 1 Department of Electrical Engineering and Computer Science, University of Maryland Baltimore County, Baltimore, MD 21250, USA; 2 Department of Electrical Engineering, Santa Clara University, Santa Clara, CA 95053, USA; 3 The Hilltop Institute, University of Maryland Baltimore County, Baltimore, MD 21250, USA; 4 Department of Diagnostic Radiology and Nuclear Medicine, University of Maryland Baltimore, Baltimore, MD 21201, USA

**Keywords:** brain connectivity, energy landscape, fMRI, resting state network, machine learning

## Abstract

The study of brain connectivity plays an important role in understanding the functional organizations of the brain. It also helps to identify connectivity signatures that can be used for evaluating neural disorders and monitoring treatment efficacy. In this work, age-related changes in brain connectivity are studied to obtain aging signatures based on various modeling techniques. These include an energy-based machine learning technique to identify brain network interaction differences between two age groups with a large (30 years) age gap between them. Disconnectivity graphs and activation maps of the seven prominent resting-state networks (RSN) were obtained from functional MRI data of old and young adult subjects. Two-sample *t*-tests were performed on the local minimums with Bonferroni correction to control the family-wise error rate. These local minimums are connectivity states showing not only which brain regions but also how strong they are working together. They work as aging signatures that can be used to differentiate young and old groups. We found that the attention network’s connectivity signature is a state with all the regions working together and young subjects have a stronger average connectivity among these regions. We have also found a common pattern between young and old subjects where the left and right brain regions of the frontal network are sometimes working separately instead of together. In summary, in this work, we combined machine learning and statistical approaches to extract connectivity signatures, which can be utilized to distinguish aging brains and monitor possible treatment efficacy.

## 1. Introduction

Research has shown that aging involves structural and functional changes in multiple brain regions, such as a decrease in the grey matter volume [1,2] and evidence of the cognitive decline in terms of memory [3], executive function, attention, and processing speed [4,5]. This cognitive decline is also associated with the integrity of the white matter fiber bundles [6,7]. Changes to the healthy aging brain networks’ complex functional and structural reconfiguration in terms of their segregation and integration make sense as a compensatory mechanism against cognitive loss associated with the aging process [8,9]. On the other hand, neuroimaging studies have revealed the importance of investigating the brain as a network. Brain regions are not functioning independently, instead they form different networks and communicate among different areas, which enables complex cognitive functioning. Hence, age-related changes in the network structure might ultimately alter functional connectivity and influence cognitive functioning. For example, Crowell, C.A., et al. in their work reported “age-related increase in network integration was driven by increases in right hemispheric connectivity to both left and right cortical regions” [10]. The authors also stated that “Older adults with higher working memory capacity demonstrated significantly higher levels of network integration in the most difficult condition” [10]. Some other findings from Moezzi, Bahar, et al. suggest “a decrease in connectivity in key networks and frequency bands associated with attention and awareness, and an increase in connectivity of the Sensorimotor (SMN) with aging during a resting state” [11]. Nearly every primary sensory and cognitive network faces some degree of age-related decline, from reduced within-network connectivity: auditory (AUD), default mode (DMN), frontoparietal (FPN), cingulo-opercular (CON), dorsal attention (ATN), and salience (SAN) networks; higher participation coefficient: SMN, visual (VIS), DMN, FPN, and ventral ATN networks; or reduced local efficiency: VIS [12].

Machine learning techniques have been used to classify or characterize functional connectivity long in the past [13,14,15], however, they are mostly pattern-related classification methods. In this work, we use energy-based machine learning techniques using maximum likelihood estimation (MLE) to obtain energy landscape and extract local minimums as connectivity state signatures. With this method, we can monitor how the brain dynamically migrates from one local minimum to the other. We also can quantitatively evaluate the connection strength with the given energy of each connectivity state. In this method, multivariate time-series data at specified regions of interest (ROIs) are obtained from the fMRI measurement. This method has been used to successfully analyze resting-state brain networks [16] and to study the brain network dynamics in high-functioning individuals with autism [17].

In this research we investigate the effects of aging on the seven prominent resting state brain networks: DMN, FPN, SMN, SAN, ATN, VIS, and AUD network. We used statistical methods like a two-sample *t*-test [18] to find the age signatures that can be used to differentiate young and old brains. These signatures could be part of the aging process and thus they can help to identify potential disorders related to aging.

## 2. Materials and Methods

### 2.1. Data and Participants

The dataset used for this study is subjects of normal aging which included 70 participants with normal cognitive function [19]. Exclusion criteria were major central nervous system (CNS) trauma, previous brain surgery, or prior documented history of stroke that resulted in lasting sequelae, active neurological dysfunction, or the use of antipsychotic and/or antiepileptic medications with known neurological side effects. The young adult group had 23 participants (aged 18–38 years; 11 male and 12 female). The old adult group included 47 participants (aged 65–90 years; 22 male and 25 female). All subjects completed and signed a consent form, which was approved by the Institutional Review Boards at Virginia Tech and the Wake Forest University School of Medicine. MR data was collected on a GE 1.5 T scanner with an eight-channel head coil. T1-weighted and rs-fMR images were collected for each subject [20].

### 2.2. Preprocessing

The data used for this study were preprocessed by using advanced connectivity analysis [21]. We first generated a brain parcellation in the native T1 space. For resting-state fMR data, preprocessing included motion correction, B0 field unwarping, slice-timing correction, skull stripping, and regression against the motion parameter time courses. Then, we registered the base fMR volume to the subject’s T1-weighted MR images. Using the deformation field generated in the registration step, an fMR-space automated anatomical labeling (AAL) parcellation of 90 ROIs was generated. After fMR space parcellation, for each AAL structure, the average time series for each subject was computed [22].

The preprocessed data was then divided into the seven RSNs based on their subnetwork affiliation using the AAL atlas [23]. The method of pairwise MEM has been applied to resting-state fMRI signals, which were obtained from the brain regions belonging to the DMN, FPN, SMN, SAN, ATN, VIS and AUD network, the 7 networks representing the RSNs.

### 2.3. Maximum Entropy Model

To analyze resting-state activity in the brain, the concept of the pairwise maximum entropy method (MEM) estimation approach described in previous studies [24,25,26] has been utilized in this study. The estimation process consists of a step for defining brain states, a pairwise MEM model for state dynamics, and an optimization step for MEM model parameters to fit network analysis of brain state transition probability distributions of empirical brain states and states generated by the model. The algorithm used to estimate the MEM is maximum likelihood estimation.

The fMRI signals at the ROIs, result in a multivariate time series. The number of ROIs is denoted by *N*. Then, the binarization is performed at each time point (i.e., in each image volume) for the fMRI signal and each ROI by thresholding the signal. A sequence of binarized signals representing the brain activity for ROI *i* (*i* = 1, *…*, *N*) is obtained {*σ_i_*(1), *…*, *σ_i_* (*t*_max_)}, where *t*_max_ is the length of the data, *σ_i_*(*t*) = 1 (*t* = 1, *…*, *t*_max_) indicates that the *i*th ROI is active at time *t*, and *σ_i_*(*t*) = −1 indicates that the ROI is inactive. The threshold is arbitrary, and is set to the time average of *σ_i_*(*t*) for each *i*. The activity pattern of the entire network at time *t* is given by an *N*-dimensional vector ***σ*** ≡ (*σ*1, *…*, *σN*) ∈ {−1, 1}*N*, where t is suppressed. There are 2*^N^* possible activity patterns in total. It has been previously shown that the pairwise MEM with binarized signals predicted anatomical connectivity of the brain better than other functional connectivity methods that are based on non-binarized continuous fMRI signals and that ternary as opposed to binary quantization did not help to improve the results [16,24]. The relative frequency, P_empirical_(σ) is with which each activity pattern is shown. The Boltzmann distribution and energy as per the authors Ezaki T et al., and Yeh, Fang-Chin et al. [19,27] is given by and is fit to P_empirical_(σ)

$$P\left(\sigma |h,\;J\right)=\frac{\exp\left[-E\left(\sigma |h,\;J\right)\right]}{\sum_{\sigma^{\prime }}\exp{\left[-E(\sigma^{\prime }|h,\;J)\right]}^{\prime }}$$

where,

$$E\left(\sigma |h,\;J\right)=-\overset{N}{\underset{i=1}{\sum }}h_{i}\sigma_{i}-\frac{1}{2}\overset{N}{\underset{i=1}{\sum }}\overset{N}{\underset{\begin{array}{c} j=1\\ j\neq i \end{array}}{\sum }}J_{ij}\sigma_{i}\sigma_{j}$$

is the energy, *h* = {*h_i_*} and ***J*** = {*J_ij_*} (*i, j = 1, …, N*) are the parameters of the model. This equation implies that, if h_i_ is large, the energy is smaller with *σ_i_* = 1 than with *σ_i_* = −1, such that the ith ROI tends to be active. The assumption is *J_ij_* = *J_ji_* and *J_ii_* = 0 (*i, j = 1, …, N*). The principle of maximum entropy implies the selection of h and J such that σ_iempirical_ = σ_imodel_ and *σ_i_σ_jempirical_* = *σ_i_σ_jmodel_* (*i, j = 1, …, N*), where empirical and model represent the expected value with respect to the empirical distribution and the model distribution, respectively. By maximizing the entropy of the distribution under these constraints, the Boltzmann distribution is given by equation,

$$P\left(\sigma |h,\;J\right)=\frac{\exp\left[-E\left(\sigma |h,\;J\right)\right]}{\sum_{\sigma^{\prime }}\exp{\left[-E(\sigma^{\prime }|h,\;J)\right]}^{\prime }}$$

This proposes that an activity pattern with a high energy value does not have a higher probability to show up and vice versa. Values of h_i_ and *J_ij_* represent the baseline activity at the ith ROI and the interaction between the *i*th and *j*th ROIs, respectively [16,24].

### 2.4. Maximum Likelihood Estimation

To estimate the MEM parameters we have used MLE, i.e., *h, **J**.* In the MLE we solve for *h, **J*** as shown below

$$\left(h,\;J\right)=\arg\underset{h,\;J}{\max}ℒ\left(h,\;J\right),$$

where ℒ(h, J) is the likelihood given by

$$ℒ\left(h,\;J\right)=\overset{t_{max}}{\underset{t=1}{\prod }}P(\sigma \left(t\right)|\;h,\;J).$$

We maximize the likelihood using a gradient ascent scheme by updating the *h, **J*** in each step [1]. Since each updating step has *2^N^* activity patterns (*N* is the number of ROIs), likelihood maximization is computationally costly for large *N*.

### 2.5. Energy Landscape Analysis

The pairwise MEM has been separately applied to all the RSNs, which are thought to be performing different roles and hence are relatively independent of each other [28,29]. There are different ROIs for the seven RSNs as per the AAL atlas: DMN has 18 ROIs, FPN has 12 ROIs, SAN has 12 RIOs, ATN has 10 ROIs, SMN has 8 ROIs, VIS has 14 ROIs, and AUD has 8 ROIs [22,23]. As discussed before the MLE has a limitation on the number of ROIs (N) we use, and because of this limitation we cannot use an ROI number greater than 12 in our energy landscape analysis using MLE. From the number of ROIs for DMN and VIS they have a large ROI (N) number. To overcome the limitation for these two networks we took the mean of left and right regions data so that we end up with 9 ROIs for DMN and 7 ROI VIS network, and further used them for the pairwise MEM method.

After the pairwise MEM is applied, the energy values at each activity state are computed using the MEM model. After that, the Dijkstra-like method is applied to the energy values to plot the disconnectivity graph and the activity pattern [16,24]. The disconnectivity graphs show the relationship between the local minimums and each leaf represents a local minimum from the activity patterns. The branching structure of the disconnectivity graph represents the energy differences among the local minimums [16,24] and each local minimum is a connectivity state with connections shown by the activity pattern. The Dijkstra algorithm helps to organize the disconnectivity graph and find out neighboring relationships from each of the state transitions in the fMRI time course. In the graph the *y*-axis shows the energy of the local minimums, and the *x*-axis shows the corresponding state number. The energy of each local minimum is proportional to the probability that a particular connectivity state has been visited. Therefore, the deeper the leaf of the disconnectivity graph (lower the energy) the higher the probability that state has been visited.

### 2.6. Statistical Analysis

A further statistical analysis was conducted on the dataset using a two-sample *t*-test [18], which is a statistical test using the sample means and sample variances of two sets of data to determine how significant the difference is between the two groups. In this case it is the brain connectivity difference between young and old subjects. To calculate the t-value and *p*-value we first obtain the sample distributions of the energy of a specified connectivity state from the two groups of subjects using the energy landscape analysis. A small *p*-value indicates the evidence against the null hypothesis. If the *p*-value is less than 0.05 that means the connectivity difference between the young and old subjects is statistically significant, and that brain connectivity state can be a potential connectivity signature.

In this study we have also performed Bonferroni correction due to multiple comparisons. While the given α (significance level) value may be appropriate for a single comparison, it is not recommended for multiple comparisons. To avoid a lot of counterfeit values, the α needs to be lowered to account for the number of comparisons being performed. To conduct that, we must obtain the total number of states for all the networks for both young and old subjects and divide the α value by this total number of states (tests). In this case the α value we have taken is 0.05, after Bonferroni correction the significance level changes to 0.05/M for each test, where “M” is the total number of comparisons, in this case it will be the total number of local minimums for all the networks.

In summary, the procedure for this study can be summed up follows: (1) In the first step the ROIs are identified based on an atlas; in this study we used AAL atlas. (2) The second step is data collection; fMRI signals are measured at the specified ROIs (from the first step). (3) In the third step the fMRI signals at each ROI are binarized to either 1 (active) or −1 (inactive) based on a threshold value. (4) In the fourth step, the relative frequency (P_empirical_(σ)) for each binarized activity pattern is computed. (5) In the fifth step the pairwise MEM model is fitted to the empirical probability distribution of all the possible activity patterns (2*^N^*, where *N* is the number of ROIs) and energy of each activity pattern is computed. (6) In the sixth step, disconnectivity graph and activity pattern are plotted. (7) Two-sample *t*-test was performed on the local minima states whose energy difference between the subject groups was high. Used Bonferroni correction on the obtained *p*-values and the connectivity states which satisfy the Bonferroni correction are reported as the potential connectivity signatures to separate the subject groups.

## 3. Results

We bundled the seven prominent brain networks, namely, DMN, FPN, ATN, SAN, SSM, AUD, and VIS networks following the AAL’s region and network assignments for the different data groups. The disconnectivity graphs and activity patterns were obtained using energy landscape analysis for the average data of 23 young subjects and 47 old subjects. The average of each group was obtained by concatenating the subject’s data for that group. In this way for each RSN, energy local minimums from both groups were noted. The local minimum states (from the activity pattern) with the higher energy difference between the two groups were considered potential connectivity signatures. These signatures were further analyzed using the two-sample *t*-test. To carry out the Bonferroni correction, we need to obtain the total number of states that are being considered in the statistical analysis. From the energy landscape results of the averaged young and old subjects’ data we found all the local minima states observed from the activity patterns for each of the seven RSNs, which are shown in Table 1.

From Table 1, there are a total of 125 local minima states for all the seven RSNs for young and old subjects combined. For the Bonferroni correction, the total number of comparisons M will be 125. Therefore, the α value is $\frac{0.05}{125}=0.0004$, which is nothing, but the Bonferroni corrected *p*-value which we will be using for finding the signatures.

**Figure 1:**
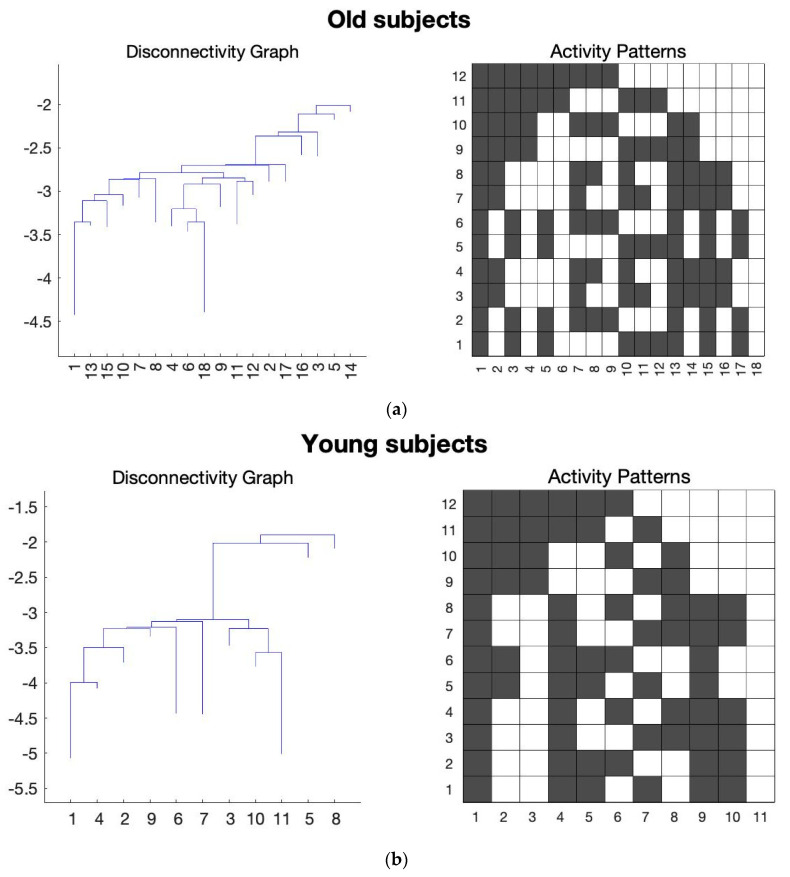
The average of all the subjects for the FPN was taken and the energy landscape analysis was conducted to generate the disconnectivity graph and activity pattern. Here, the *x*-axis of the disconnectivity graph represents the local minima number and *y*-axis shows its corresponding energy value. For the activity patterns, *x*-axis is the local minima number and *y*-axis shows the ROIs for FPN. (**a**) Energy landscape analysis conducted on the average of the old subjects for FPN. (**b**) Energy landscape analysis conducted on the average of the young subjects for FPN.

**Figure 2:**
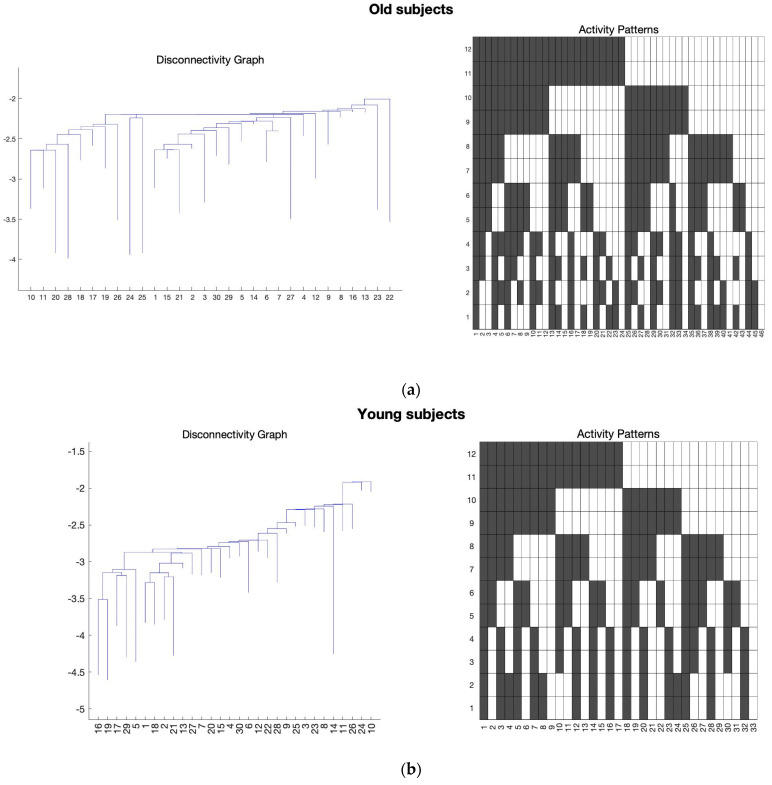
The average of all the subjects for the SAN was taken and the energy landscape analysis was conducted to generate the disconnectivity graph and activity pattern. Here, the *x*-axis of the disconnectivity graph represents the local minima number and *y*-axis shows its corresponding energy value. For the activity patterns, *x*-axis is the local minima number and *y*-axis shows the ROIs for SAN. (**a**) Energy landscape analysis conducted on the average of the old subjects for SAN. (**b**) Energy landscape analysis conducted on the average of the young subjects for SAN.

**Figure 3:**
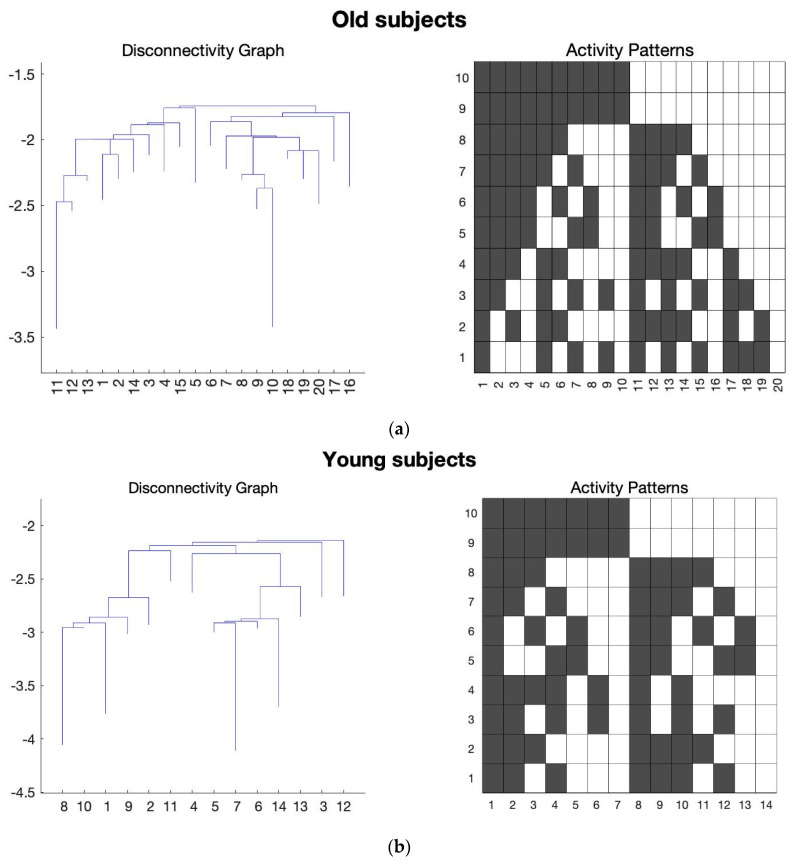
The average of all the subjects for the ATN was taken and the energy landscape analysis was conducted to generate the disconnectivity graph and activity pattern. Here, the *x*-axis of the disconnectivity graph represents the local minima number and *y*-axis shows its corresponding energy value. For the activity patterns, *x*-axis is the local minima number and *y*-axis shows the ROIs for ATN. (**a**) Energy landscape analysis conducted on the average of the old subjects for ATN. (**b**) Energy landscape analysis conducted on the average of the young subjects for ATN.

After performing the Bonferroni correction, we found several states in FPN, SAN, and ATN networks that can satisfy the Bonferroni correction, and they can be considered as potential signatures for distinguishing aging brains. Figure 1, Figure 2 and Figure 3 show the average results of the old and young subjects for these networks, respectively.

In the Figure 1, Figure 2 and Figure 3, the *y*-axis of the activity patterns refers to the ROIs for the corresponding RSN. Each RSN has different number of ROIs which have been assigned based on the AAL atlas [22,23]. Table 2, Table 3 and Table 4 show the ROIs for FPN, SAN, and the ATN network, respectively.

**Figure 4:**
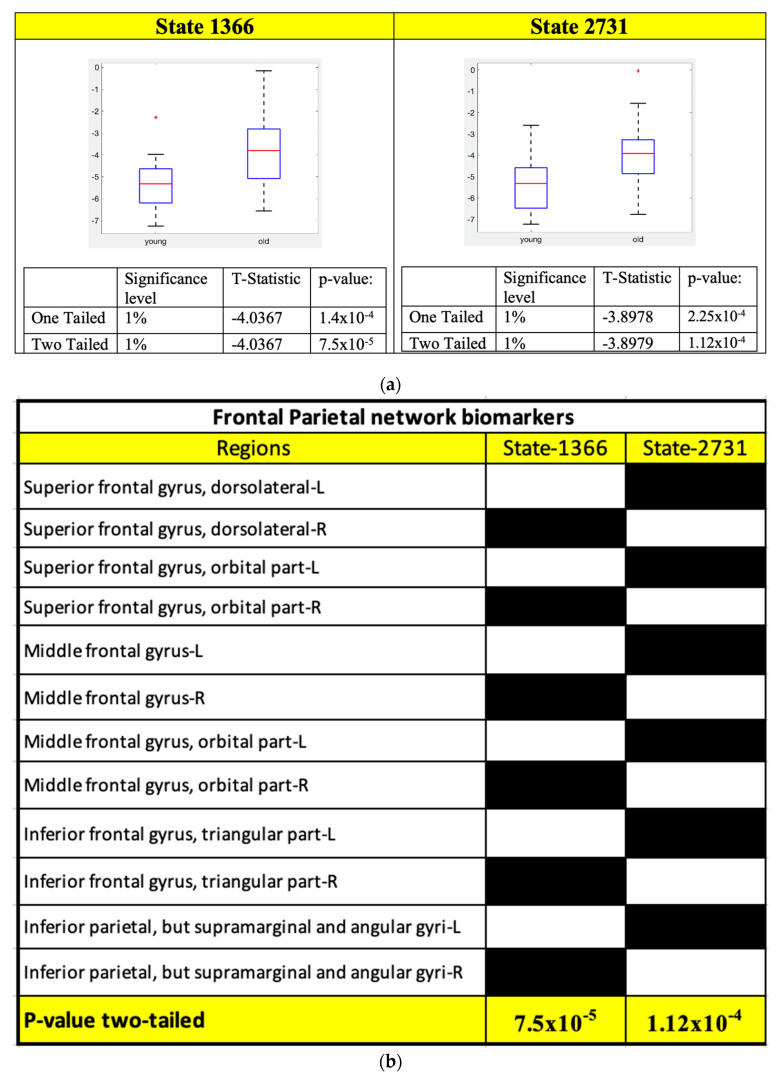
Two–sample *t*-test was performed on the local minimum states of the FPN whose energy difference was maximum and the states whose *p*–values satisfy the Bonferroni correction are considered potential connectivity signatures. (**a**) Two–sample *t*-test results of the connectivity signature state which satisfy the Bonferroni correction for FPN. (**b**) Connectivity signature states definitions for FPN.

**Figure 5:**
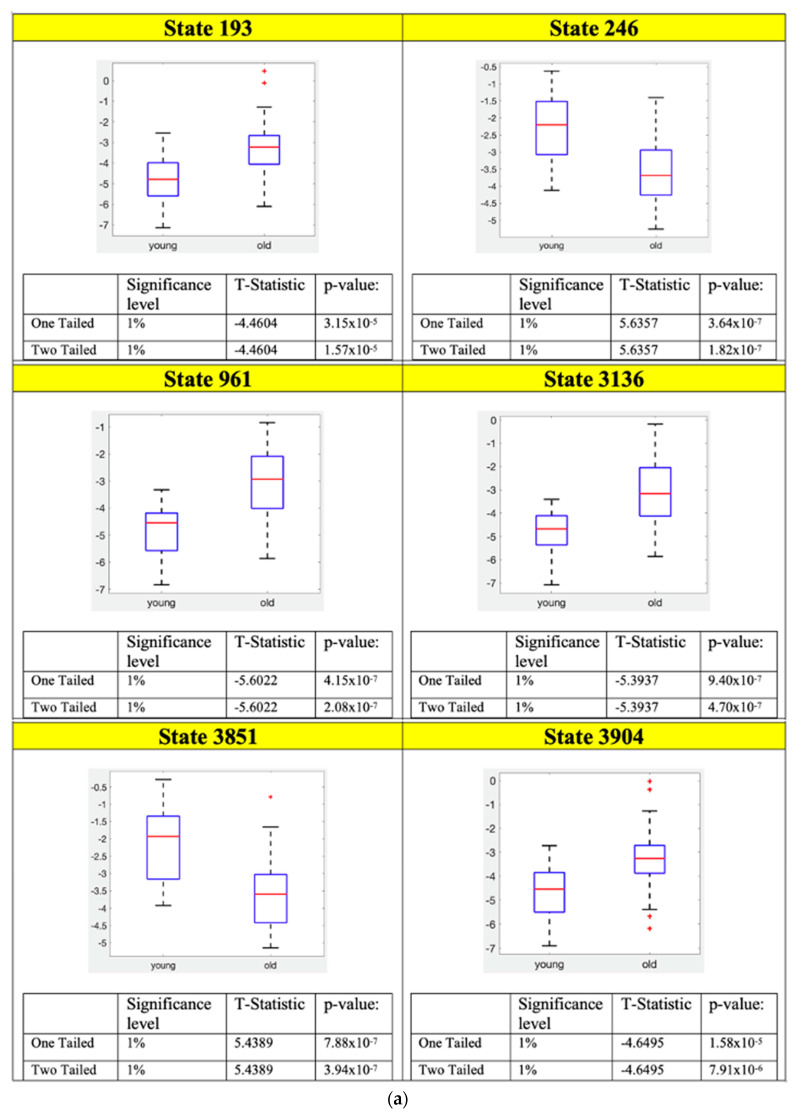

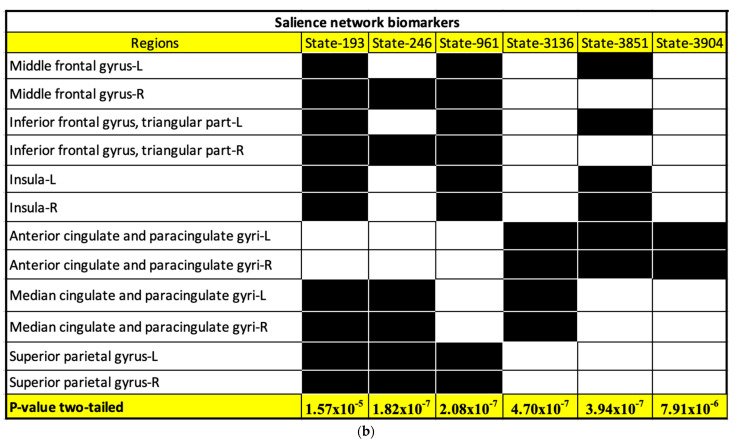
Two–sample *t*-test was performed on the local minimum states of the SAN whose energy difference was maximum and the states whose *p*–values satisfy the Bonferroni correction are considered potential connectivity signatures. (**a**) Two–sample *t*-test results of the connectivity signature state for SAN. (**b**) Connectivity signature states definitions for SAN.

**Figure 6:**
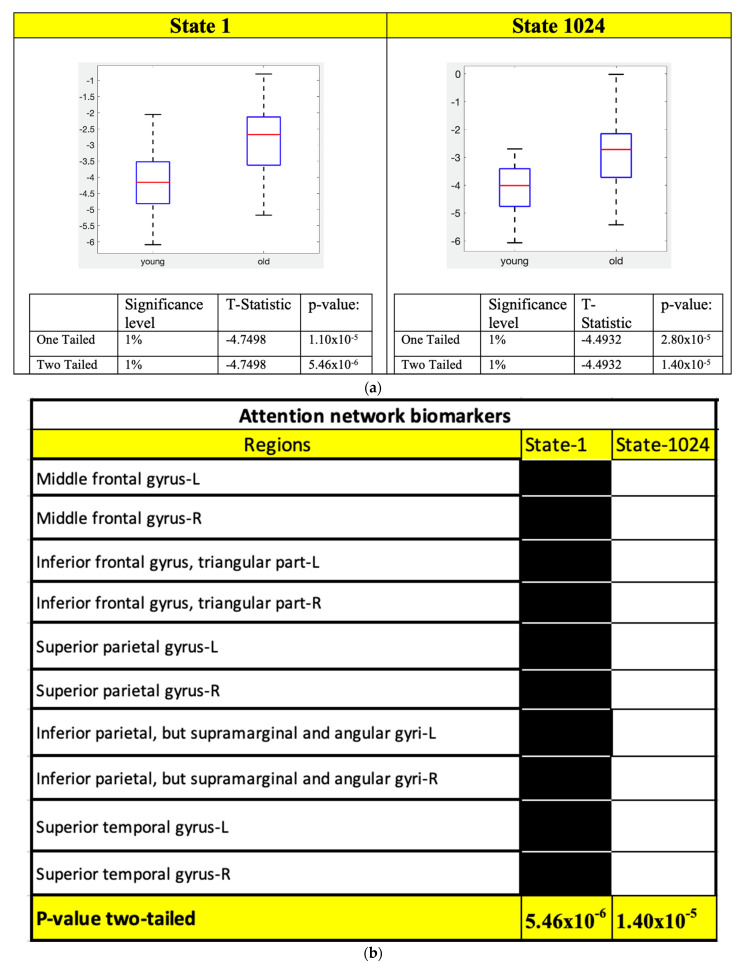
Two–sample *t*-test was performed on the local minimum states of the ATN whose energy difference was maximum and the states whose *p*–values satisfy the Bonferroni correction are considered potential connectivity signatures. (**a**) Two–sample *t*-test results of the connectivity signature states for ATN. (**b**) Connectivity signature states definitions for ATN and ROIs functionality are shown to help interpret the meaning of these connectivity signatures.

Two-sample *t*-test results for the FPN, SAN and ATN states and their corresponding ROIs connectivity are shown in Figure 4, Figure 5 and Figure 6, respectively.

The following conclusions can be drawn from the functionality of the individual ROIs for SAN, ATN, and FPN networks. Figure 4a shows that there are two states in FPN which are statistically significant and satisfy Bonferroni correction. Both states’ ROI connectivity show the left and right parts of the FPN are working independently (Figure 4b). Furthermore, for both states, young subjects have much lower energies than that of old subjects (Figure 4a). That means for young subjects their left and right sides in FPN are working more separately since lower energy states are those visited more frequently and ROIs in them are more strongly connected.

Similar behavior can be observed in SAN as shown in Figure 5b, where the frontal part of the states 246 and 3851 are showing that the left and right regions are again working separately. In Figure 5b, it also shows that the state 246 insula is working together with the left part of the frontal gyrus and from the box plots we can see that this behavior is more prominent in the old subjects for the SAN network. In addition, the state 3851, it is seen only working with the right part of the frontal gyrus and for this state young subjects have much lower energy as per the box plot results from Figure 5a. State 193 and 3904 in SAN have anterior cingulate and paracingulate gyri working independently from the rest of the ROIs in SAN.

Figure 6b shows that the only states in the ATN network that can separate the young and old subjects are the states that all ATN ROIs are working together, and from the box plots in Figure 6a, the energy of these states is lower for young subjects. It implies that in the ATN young subjects have stronger connectivity compared that of the old subjects.

To compare these new findings with previous reported studies, while several past studies have noted modularity effects on aging [30,31,32] and intra-network connections at rest [32], the current study didn’t focus on these metrics. We did replicate the results from past studies showing the reduction in the intra-network connectivity due to aging [30,31,32].

## 4. Discussion

We have used an energy-based machine learning technique to analyze and identify the brain connectivity differences between the subjects of two age groups with a large age gap. The maximum likelihood estimation method is used to estimate the MEM model parameters h, **J**. The MEM enables us to create an energy-based model which we use to generate disconnectivity graphs and activity patterns, which helps us to map the structure of the brain connectivity states of the seven prominent RSNs. The activity patterns show the local minimums whose energy is smaller than that of its neighbors; the deeper the local minima, the more frequently the connectivity state is visited. Since the MLE estimates the model parameters by updating h, **J** for each iteration, each update step involves 2*^N^* activity patterns to calculate, where N would be the total number of ROIs. Therefore, the likelihood estimation becomes computationally costly for larger number of ROIs (N).

Using the statistical analysis on the local minima connectivity states we found that few of the connectivity states have significantly low energy values and satisfy the Bonferroni correction. These are considered as potential signatures for differentiating the aging brain. The RSNs whose connectivity states satisfied the Bonferroni correction are the FPN, SAN, and ATN networks and Figure 1, Figure 2 and Figure 3 show the disconnectivity graphs and activity patterns for these RSNs, respectively. Based on the box plots for FPN (Figure 4a), we could say that functional connectivity among the FPN ROIs decreases with aging as in several studies which showed similar results [33,34,35,36]. In this study, we found a new ROI connectivity for FPN where we noticed that the left and right parts of the brains are working independently for the ROIs, which means if all the left ROIs are active, the corresponding right ROIs are inactive, and vice versa. Furthermore, looking at the box plot for FPN (Figure 4a), we can see that for old subjects that particular connectivity states have higher energies compared to that of young subjects, meaning that the connectivity strength among FPN ROIs is decreasing due to aging.

The behavior of the left and right parts of the brains working independently in the frontal part is also observed in the states 246 and 3851 of the connectivity states in SAN. Figure 5b shows the connectivity states definitions for these states. The ROIs one and two of the SAN which are middle frontal gyrus—L and middle frontal gyrus—R are seen working independently. There is also another connectivity bundling observed here which is the insula region working with the left part of the brain within the SAN. For this connectivity state (3851), old subjects have much lower energy compared to the young subjects indicating a unique connectivity pair prominent in old subjects where the insula region is seen working with the left side of middle frontal gyri. In addition, for this connectivity we see that the superior parietal region is working with the right part of the frontal gyrus. Therefore, we see a unique connectivity between the insular region and the frontal cortex. There are studies in the past which reported a decrease in the functional connectivity of the right fronto-insular cortex with the DMN and the FPN [37,38], but our focus in this study was mainly the intra-network connectivity. Thus, this is possible work for the future to see the inter-network connectivity between the two age groups of subjects. Another connectivity state we observed was the anterior cingulate cortex working against the rest of the ROIs in the SAN. The anterior cingulate cortex is involved in linking reward and punishment information, which trigger emotional reactions, to behavior and to actions [39,40,41]. Therefore, a possible interpretation of this connectivity state could be that the regions responsible for emotional processing can work independently from the rest of the ROIs in the SAN network, and from the box plots, we can see this behavior is more prominent in the young subjects.

Moreover, comparing all the connectivity signatures we found for the SAN (Figure 5b) most of the states show a decreased connectivity with aging, similar to the results from other studies using different techniques [42]. For the ATN, the only connectivity states which satisfy the Bonferroni correction are the states 1 and 1024 (Figure 6b), and we were able to replicate the results from similar studies showing a decrease in the connectivity with age [43].

The major limitation in this study using the MLE technique for estimating the MEM parameters would be computational cost for larger ROIs as discussed. For this study, the highest number of ROIs are observed for DMN and VIS networks with 18 and 14 ROIs, respectively. For these two networks the energy calculations were computationally challenging, so to reduce these challenges we have taken the average of the left and right regions for each ROI data and we used them for the energy calculations.

## 5. Conclusions and Future Work

In this study, by applying energy-based machine learning techniques using maximum likelihood estimation we obtained energy landscapes of the dataset and extracted local minimums as connectivity state signatures. We have observed the brain intra-network connectivity differences between young and old subjects and, based on the two-sample *t*-test results, we found that the RSNs, FPN, SAN, and ATN show a decrease in the connectivity with age.

The underlined limitation for this method, as discussed above, is the exponential increase in the computation power with the increase in the ROI number. However, this method has shown to quickly identify the connectivity signature of the different groups of data used and can quantitatively determine the energy of the visiting state so that we can separate them using the distribution of the energies based on the *p*-values.

Further research is needed to add more ROIs to the energy-based machine learning approach used to identify functional connectivity signatures. This needs further investigation and search for a possible solution so that this approach can be more effectively used to understand more focused functional connectivity of the human brain. The dataset used for this study was only imaging data without any behavioral data, limiting the interpretation of results purely based on the network connectivity rather than based on the functionality of the ROIs. In future studies using this method, it will be useful to have behavioral data for a better interpretation of results.

## Figures and Tables

**Table 1:** Total number of local minimums for each RSN.

Resting State Network	Total Number of Local Minimums for Old and Young Subjects
DMN	11
FPN	21
SMN	7
SAN	52
ATN	24
VIS	4
AUD	6

**Table 2:** ROIs of the FPN corresponding to the activity pattern in Figure 1a,b.

Frontoparietal Network	
Node Number	Region
1	Superior frontal gyrus, dorsolateral-L
2	Superior frontal gyrus, dorsolateral-R
3	Superior frontal gyrus, orbital part-L
4	Superior frontal gyrus, orbital part-R
5	Middle frontal gyrus-L
6	Middle frontal gyrus-R
7	Middle frontal gyrus, orbital part-L
8	Middle frontal gyrus, orbital part-R
9	Inferior frontal gyrus, triangular part-L
10	Inferior frontal gyrus, triangular part-R
11	Inferior parietal, but supramarginal and angular gyri-L
12	Inferior parietal, but supramarginal and angular gyri-R

**Table 3:** ROIs of the SAN corresponding to the activity pattern in Figure 2a,b.

Salience Network	
Node Number	Region
1	Middle frontal gyrus-L
2	Middle frontal gyrus-R
3	Inferior frontal gyrus, triangular part-L
4	Inferior frontal gyrus, triangular part-R
5	Insula-L
6	Insula-R
7	Anterior cingulate and paracingulate gyri-L
8	Anterior cingulate and paracingulate gyri-R
9	Median cingulate and paracingulate gyri-L
10	Median cingulate and paracingulate gyri-R
11	Superior parietal gyrus-L
12	Superior parietal gyrus-R

**Table 4:** ROIs of the ATN corresponding to the activity pattern in Figure 3a,b.

Attention Network	
Node Number	Region
1	Middle frontal gyrus-L
2	Middle frontal gyrus-R
3	Inferior frontal gyrus, triangular part-L
4	Inferior frontal gyrus, triangular part-R
5	Superior parietal gyrus-L
6	Superior parietal gyrus-R
7	Inferior parietal, but supramarginal and angular gyri-L
8	Inferior parietal, but supramarginal and angular gyri-R
9	Superior temporal gyrus-L
10	Superior temporal gyrus-R

## Data Availability

The data supporting the findings of this study are available within the article and its supplementary materials. Raw data that support the findings of this study are available upon request from the corresponding author.

## Supplementary Materials

The following supporting information can be downloaded at: https://www.mdpi.com/article/10.3390/s23031603/s1.
