# Supplementary material for: Age Related Functional Connectivity Signature Extraction Using Energy-Based Machine Learning Techniques
Source: Sensors (Basel). 2023 Feb 1;23(3):1603. doi: 10.3390/s23031603 (PMC9920122; doi:10.3390/s23031603)
Supplement: Supplementary file 1 [file sensors-23-01603-s001.zip › sensors-2151576-supplementary.pdf]

sensors

MDPI

Age Related Functional Connectivity Signature Extraction Using Energy-Based Machine Learning Techniques

Material and Methods:

Maximum Entropy Method (MEM):

The fMRI signals at the ROIs result in a multivariate time series. The number of ROIs is denoted by N. Then, the binarization is performed at each time point (i.e., in each image volume) for the fMRI signal and each ROI by thresholding the signal. A sequence of binarized signals representing the brain activity for ROI i ($i = 1, \ldots, N$) is obtained $\{\sigma_i(1), \ldots, \sigma_i(t_{max})\}$, where t_{max} is the length of the data, $\sigma_i(t) = 1$ ($t = 1, \ldots, t_{max}$) indicates that the ith ROI is active at time t, and $\sigma_i(t) = -1$ indicates that the ROI is inactive. The threshold is arbitrary and is set to the time average of $\sigma_i(t)$ for each i. The activity pattern of the entire network at time t is given by an N-dimensional vector.

$\boldsymbol{\sigma} \equiv (\sigma 1, \ldots, \sigma N) \in \{-1, 1\}N$, where t is suppressed. There are 2^N possible activity patterns in total. It has been previously shown that the pairwise MEM with binarized signals predicted anatomical connectivity of the brain better than other functional connectivity methods that are based on non-binarized continuous fMRI signals and that ternary as opposed to binary quantization did not help to improve the results (Watanabe et al., 2013, Ezaki et al., 2017). The relative frequency $P_{empirical}(\sigma)$ is with which each activity pattern is shown. The Boltzmann distribution is given by and is fit to $P_{empirical}(\sigma)$.

$$P(\boldsymbol{\sigma} \mid \boldsymbol{h}, \mathbf{J}) = \frac{\exp\left[-E(\boldsymbol{\sigma} \mid \boldsymbol{h}, \mathbf{J})\right]}{\sum_{\boldsymbol{\sigma}'} \exp\left[-E(\boldsymbol{\sigma}' \mid \boldsymbol{h}, \mathbf{J})\right]},$$

where,

$$E(\boldsymbol{\sigma} \mid \boldsymbol{h}, \mathbf{J}) = -\sum_{i=1}^{N} h_i \sigma_i - \frac{1}{2} \sum_{i=1}^{N} \sum_{\substack{j=1 \\ j \neq i}}^{N} J_{ij} \sigma_i \sigma_j$$

is the energy, $h = \{h_i\}$ and $J = \{J_{ij}\}$ ($i, j = 1, \ldots, N$) are the parameters of the model. This equation implies that, if h_i is large, the energy is smaller with $\sigma_i = 1$ than with $\sigma_i = -1$, such that the ith ROI tends to be active. The assumption is $J_{ij} = J_{ji}$ and $J_{ii} = 0$ ($i, j = 1, \ldots, N$). The principle of maximum entropy implies the selection of h and J such that $\sigma_{iempirical} = \sigma_{imodel}$ and $\sigma_i\sigma_{jempirical} = \sigma_i\sigma_{jmodel}$ ($i, j = 1, \ldots, N$), where empirical and model represent the expected value with respect to the empirical distribution and the model distribution, respectively. By maximizing the entropy of the distribution under these constraints, the Boltzmann distribution is given by equation,

$$P(\boldsymbol{\sigma} \mid \boldsymbol{h}, \mathbf{J}) = \frac{\exp\left[-E(\boldsymbol{\sigma} \mid \boldsymbol{h}, \mathbf{J})\right]}{\sum_{\boldsymbol{\sigma}'} \exp\left[-E(\boldsymbol{\sigma}' \mid \boldsymbol{h}, \mathbf{J})\right]},$$

This proposes that an activity pattern with a high energy value does not have a higher probability to show up and vice versa. Values of h_i and J_{ij} represent the baseline activity at the ith ROI and the interaction between the ith and j^{th} ROIs, respectively (Ezaki et al., 2017; Watanabe et al., 2013).

To examine the energy landscape of the RSNs, binarization of the normalized signals with a threshold of 0 has been performed. At a given energy E, the minima can be classified into disjoint sets, if they are connected by pathways or intervening minima, where the energy never exceeds E. Two minima are in different sets if the highest energy transitions state (a local maxima) or lowest energy pathway between them exceeds E. If more than one path connects two minima (which is not possible in the 1D case) only the lowest energy path is considered in constructing the set. As E is raised more and more minima become accessible and eventually only one set will remain, containing all the minima

(Abraham, 2004). To visualize the relationship between the local minima of energy, a disconnectivity graph is constructed. This graph has been used to study the Ising spin model, which is equivalent to the pairwise MEM (Zhou, 2011; Zhou and Wong, 2009), (Abraham, 2004; Doye et al., 1999; Hordijk, n.d.; Lempesis et al., 2013). In reference to the spin systems, a disconnectivity graph with a continuous energy threshold, where the energy threshold is defined as (Hordijk, n.d.;) is also referred to as a barrier tree. A local minimum is a node whose energy is smaller than those of all the neighboring nodes. The disconnectivity graph is basically a plot representing the local minima. In order to obtain the disconnectivity graph, a reduced network of the local minima has been formed in which each local minimum was isolated. Disconnectivity graph is a hierarchical tree whose terminals are the local minima. The vertical position of the terminals and internal nodes of the disconnectivity graph represents an energy value (Ezaki et al., 2017).

The disconnectivity graph is obtained by the following procedures used by (Ezaki et al., 2017). First, enumerate local minimums, i.e., the activity patterns whose energy is smaller than that of all neighbors. Then, for a given pair of local minimums $\boldsymbol{\alpha}$ and $\boldsymbol{\alpha}'$, we consider a path connecting them, $\boldsymbol{\alpha}\leftrightarrow\boldsymbol{\alpha}'$, where a path is defined as a sequence of activity patterns starting from $\boldsymbol{\alpha}$ and ending at $\boldsymbol{\alpha}'$ such that any two consecutive activity patterns on the path are neighboring patterns. $E_{\max}(\boldsymbol{\alpha}\leftrightarrow\boldsymbol{\alpha}')$ is denoted by the largest energy value among the activity patterns on path $\boldsymbol{\alpha}\leftrightarrow\boldsymbol{\alpha}'$. The brain dynamics on this path must climb up the hill to go through the activity pattern with energy $E_{\max}(\boldsymbol{\alpha}\leftrightarrow\boldsymbol{\alpha}')$ to travel between $\boldsymbol{\alpha}$ and $\boldsymbol{\alpha}'$. Because a large energy value corresponds to a low frequency of the activity pattern, a large $E_{\max}(\boldsymbol{\alpha}\leftrightarrow\boldsymbol{\alpha}')$ value implies that the frequency of switching between $\boldsymbol{\alpha}$ and $\boldsymbol{\alpha}'$ along this path is low. Because various paths may connect $\boldsymbol{\alpha}$ and $\boldsymbol{\alpha}'$, it is considered $E\boldsymbol{\alpha}\boldsymbol{\alpha}' = \min_{\boldsymbol{\alpha}\leftrightarrow\boldsymbol{\alpha}'} E_{\max}(\boldsymbol{\alpha}\leftrightarrow\boldsymbol{\alpha}')$.

If all the rarest activity patterns are removed whose energy is equal to or larger than $E\boldsymbol{\alpha}\boldsymbol{\alpha}'$, then $\boldsymbol{\alpha}$ and $\boldsymbol{\alpha}'$ are disconnected which implies that no path connecting them exists. The energy barrier for the transition from $\boldsymbol{\alpha}$ to $\boldsymbol{\alpha}'$ is given by $E\boldsymbol{\alpha}\boldsymbol{\alpha}' - E(\boldsymbol{\alpha})$.

To calculate $E\boldsymbol{\alpha}\boldsymbol{\alpha}'$, a method such as Dijkstra has been employed (Ezaki et al., 2017). Considering the hypercube composed of $2N$ activity patterns. Two nodes (i.e., activity patterns) are adjacent to each other (i.e., directly connected by a link) if they are neighboring activity patterns. Each node has degree (i.e., number of neighbors) N. Then, a local minimum activity pattern $\boldsymbol{\alpha}$ is fixed and $E\boldsymbol{\alpha}\boldsymbol{\alpha}'$ is obtained for all local minimums $\boldsymbol{\alpha}'$. $E\boldsymbol{\alpha}\boldsymbol{\alpha} = E(\boldsymbol{\alpha})$ and $E\boldsymbol{\alpha}\boldsymbol{\alpha}' = E(\boldsymbol{\alpha}')$ for all $\boldsymbol{\alpha}'$ have been fixed such that neighbors of $\boldsymbol{\alpha}$. These values are finalized and cannot be changed. $E\boldsymbol{\alpha}\boldsymbol{\alpha}'$ for the other $2N - N - 1$ local minimums $\boldsymbol{\alpha}'$ are initialized to ∞. Then, this procedure iterates until $E\boldsymbol{\alpha}\boldsymbol{\alpha}'$ values for all the nodes $\boldsymbol{\alpha}'$ are finalized.

(i) For each finalized $\boldsymbol{\alpha}'$, update $E\boldsymbol{\alpha}\boldsymbol{\alpha}''$ for its all unfinalized neighbors $\boldsymbol{\alpha}''$
(ii) Find $\boldsymbol{\alpha}'$ with the smallest unfinalized $E\boldsymbol{\alpha}\boldsymbol{\alpha}'$ value and finalize it.
(iii) The first two steps are repeated. This procedure is carried out for each local minimum $\boldsymbol{\alpha}$, resulting in $E\boldsymbol{\alpha}\boldsymbol{\alpha}'$ for all pairs of local minimums. By collecting pairs of local minimums that have the same $E\boldsymbol{\alpha}\boldsymbol{\alpha}'$ value, a set of local minimums is specified that happens to be located under the same branch. The information obtained so far is enough to plot a dendrogram of local minimums, i.e., the disconnectivity graph.

Each local minimum has a basin of attraction in the state space, Ω. Each activity pattern, denoted by $\boldsymbol{\sigma}$, usually belongs to one of the attractive basins, which is determined as follows.

(i) Unless $\boldsymbol{\sigma}$ is a local minimum, move to the neighboring activity pattern that has the smallest energy value.
(ii) The first step is repeated until a local minimum, denoted by $\boldsymbol{\alpha}$, is reached. Hence, concluding that $\boldsymbol{\sigma}$ belongs to the attractive basin of $\boldsymbol{\alpha}$.
(iii) The steps (i) and (ii) are repeated for all the initial activity patterns $\boldsymbol{\sigma} \in \Omega$.

Basin Size of Local Minimum:

After the calculation of local minima, we progress to obtain the size of the basin of each local minimum as followed by (Stillinger and Weber, 1982, Zhou and Wong, 2009). At first there is an appropriate selection of a starting node i, which must be one of the $2N$ nodes in the network of network states. Then, the neighbor of node i possessing the smallest energy level is identified and denoted by j.

If $E(Vj) < E(Vi)$, it is moved to node j. This move is in accordance with the steepest descent at node i. If such a node j did not exist, it remained at node i. In the latter case, i is a local minimum. If it moved to node j, one must look for the steepest descent from node j and continue to travel until arrived at a local minimum. The starting node i belongs to the basin of the local minimum that has been finally reached. Same procedure has been performed for all i. The basin size of a local minimum is the fraction of nodes that belong to the basin of the local minimum. Brain state local minimums showing both complementary states are indicating a stable in-phase and anti-phase relationship between two groups of ROIs. Within the same group, ROIs are coherently coupled and anti-correlated to activities of ROIs in the other group.

Energy Landscape:

To describe the dynamics of the brain system at rest, the energy landscape analysis has been performed. More specifically, first, elucidation of the local minima (attractors), and then evaluation of the energy barriers between pairs of attractors has been performed, following the procedure described in previous works (Ezaki et al., 2017; Watanabe et al., 2013, 2014; Watanabe and Rees, 2017). To construct an energy landscape, the distance between two states should be first defined. Based on this distance, neighbor states are defined to extract local minima. The local minima (also called stable states) are defined as states that have lower energy (more frequent) relative to their neighbors. To evaluate the energy barrier for each local minima pair, the lowest energy pathways must be extracted by using disconnectivity graph analysis discussed above. Specifically, for each possible pair of local minima, the shortest path connecting the two local minima has been recorded. The highest energy on this path has been selected as a threshold to remove states that exhibited higher energy than the threshold. The highest energy value of the last connected path has been assigned to the threshold of the local minimum pair (Kang et al., 2019). The disconnectivity graph and activity pattern for DMN, SMN, VIS, and AUD network are shown in **Figures S1, S2, S3,** and **S4,** respectively. The disconnectivity graph and activity pattern for FPN, SAN, and ATN network can be seen in **Figures 1, 2, and 3,** respectively, of the main text. Here the x-axis of the disconnectivity graph represents the local minima state number and y-axis shows its corresponding energy value. For the activity patterns, x-axis is the local minima state number and y-axis shows the ROIs of that particular RSN.

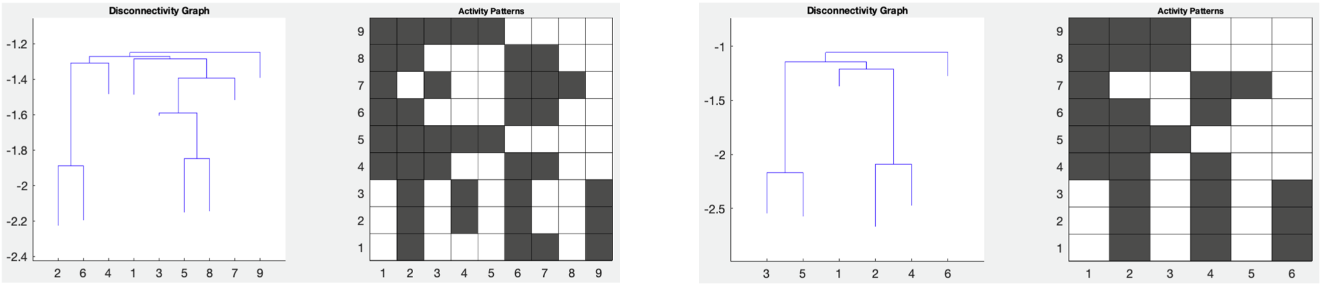

(**a**) Old subjects average (**b**) Young subjects average

Figure S1. The average of all the subjects for the DMN was taken and the energy landscape analysis was conducted to generate the disconnectivity graph and activity pattern. (**a**) Energy landscape analysis conducted on the average of the old subjects. (**b**) Energy landscape analysis conducted on the average of the young subjects.

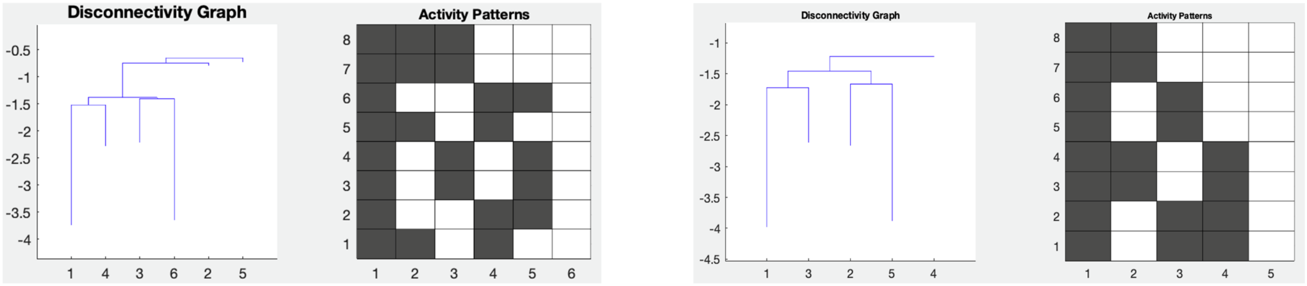

(**a**) Old subjects average (**b**) Young subjects average

Figure S2. The average of all the subjects for the SMN was taken and the energy landscape analysis was conducted to generate the disconnectivity graph and activity pattern. (**a**) Energy landscape analysis conducted on the average of the old subjects. (**b**) Energy landscape analysis conducted on the average of the young subjects.

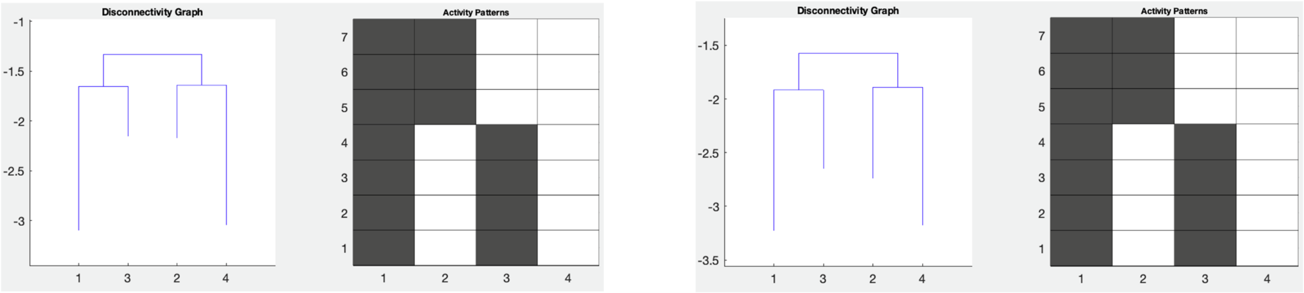

(**a**) Old subjects average (**b**) Young subjects average

Figure S3. The average of all the subjects for the VIS was taken and the energy landscape analysis was conducted to generate the disconnectivity graph and activity pattern. (**a**) Energy landscape analysis conducted on the average of the old subjects. (**b**) Energy landscape analysis conducted on the average of the young subjects.

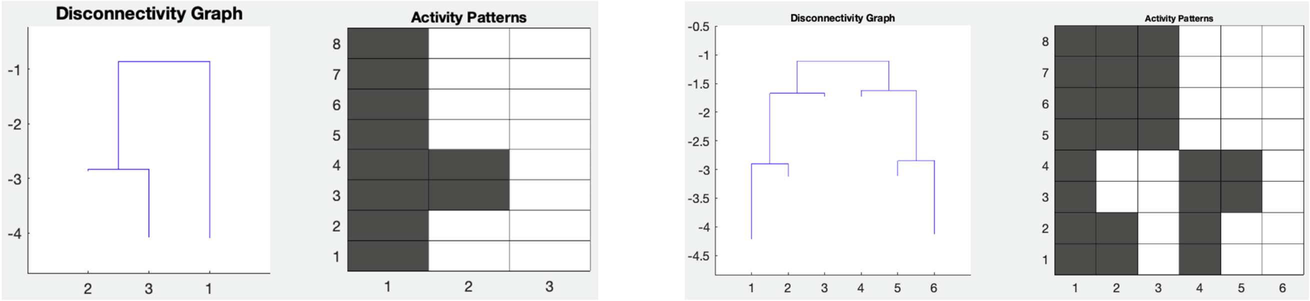

(**a**) Old subjects average (**b**) Young subjects average

Figure S4. The average of all the subjects for the AUD was taken and the energy landscape analysis was conducted to generate the disconnectivity graph and activity pattern. (**a**) Energy landscape analysis conducted on the average of the old subjects. (**b**) Energy landscape analysis conducted on the average of the young subjects.

The **Tables S1, S2, S3,** and **S4** show the ROIs of the networks corresponding to the activity pattern in **Figure S1, S2, S3, and S4,** respectively.

Table S1. ROIs of the DMN corresponding to the activity pattern in **Figure S1.**

DMN	
1	Superior frontal gyrus, medial-L,R
2	Superior frontal gyrus, medial orbital-L,R
3	Anterior cingulate and paracingulate gyri-L,R
4	Posterior cingulate gyrus-L,R
5	Para hippocampal gyrus-L,R
6	Angular gyrus-L,R
7	Precuneus-L,R
8	Middle temporal gyrus-L,R
9	Temporal pole: middle temporal gyrus-L,R

Table S2. ROIs of the SMN corresponding to the activity pattern in **Figure S2.**

SMN	
1	Supplementary motor area-L
2	Middle frontal gyrus-R
3	Inferior frontal gyrus, triangular part-L
4	Inferior frontal gyrus, triangular part-R

5	Superior parietal gyrus-L
6	Superior parietal gyrus-R
7	Inferior parietal, but supramarginal and angular gyri-L
8	Inferior parietal, but supramarginal and angular gyri-R

Table S3. ROIs of the VIS corresponding to the activity pattern in **Figure S3.**

VIS	
1	Calcarine fissure and surrounding cortex-L,R
2	Cuneus-L,R
3	Lingual gyrus-L,R
4	Superior occipital gyrus-L,R
5	Middle occipital gyrus-L,R
6	Inferior occipital gyrus-L,R
7	Fusiform gyrus-L,R

Table S4. ROIs of the AUD corresponding to the activity pattern in **Figure S4.**

AUD	
1	Rolandic operculum-L
2	Rolandic operculum-R
3	Supramarginal gyrus-L
4	Supramarginal gyrus-R
5	Heschl gyrus-L
6	Heschl gyrus-R
7	Superior temporal gyrus-L
8	Superior temporal gyrus-R

Two-sample t-test calculations and results:

Before performing the two-sample t-test, the energy differences between young and old subjects of all the possible local minimums for the seven ROIs are noted. The states whose energy differences are the highest are considered as potential connectivity signatures and t-test results calculated on these connectivity signatures.

The Tables S5, S6, S7, S8, S9, S10, and S11 show the energy values of all the local minima states from the young and old subject's average results for DMN, FPN, SAN, ATN, SMN, VIS, and AUD network, respectively.

Table S5. Energy values of all the states for young and old subjects' average results for DMN network.

Local Minima Index	State Number	Old Energy	Young Energy	Difference
1-young and old	8	−1.48476421	−1.365305917	0.11945829
2-young and old	65	−2.226868058	−2.664250258	−0.4373822
3-young	112	−2.090757252	−2.546942418	−0.4561852
3-old	168	−1.602049297	−1.058014478	0.54403482
4-old	234	−1.482665306	−1.284692867	0.19797244
5-old	240	−2.14841088	−2.186345516	−0.0379346
6-old	273	−2.194920935	−2.090776081	0.10414485
7-old	279	−1.519166781	−1.244905942	0.27426084
4-young	401	−2.156496588	−2.472472086	−0.3159755

8-old, 5-young	448	−2.14641894	−2.575675447	−0.4292565
9-old, 6-young	505	−1.392102963	−1.278617914	0.11348505

Table S6. Energy values of all the states for young and old subjects' average results for FPN network.

Local Minima Index	State Number	Old Energy	Young Energy	Difference
	1	−4.418887722	−5.064319792	−0.6454321
	52	−2.894885699	−2.964718482	−0.0698328
	205	−2.595612003	−2.686468831	−0.0908568
2-young	208	−2.708183431	−3.712541761	−1.0043583
	256	−3.403756353	−3.468913044	−0.0651567
4-young	769	−3.211832947	−4.077628405	−0.8657955
	973	−2.179203022	−2.226766478	−0.0475635
	1024	−3.468512934	−3.002031143	0.46648179
	1298	−3.076435394	−3.449900591	−0.3734652
6-young, 8-old	1366	−3.357065708	−4.436092196	−1.0790265
	1502	−3.18260247	−3.300828497	−0.118226
	2595	−3.173234783	−3.281729781	−0.108495
7-young, 11-old	2731	−3.379830609	−4.447981028	−1.0681504
	2799	−3.050196692	−3.428908668	−0.378712
	3073	−3.397673918	−2.794468606	0.60320531
	3124	−2.089095033	−2.092156032	−0.003061
	3841	−3.415959492	−3.346351804	0.06960769
10-young	3889	−2.821050341	−3.772270035	−0.9143671
	3892	−2.588546169	−2.636859683	0.27528463
	4045	−2.890217822	−2.840264035	−0.1344645
	4096	−4.394950873	−5.012817437	−0.5400886

Table S7. Energy values of all the states for young and old subjects' average results for SAN network.

Local Minima Index	State Number	Old Energy	Young Energy	Difference
	1	−3.110415099	−3.827590109	0.717175009
	4	−2.623843827	−2.393447467	0.230396359
	16	−3.295090906	−3.791496592	0.496405685
	49	−2.461678124	−2.509145577	0.047467453
	54	−2.535105364	−2.186321015	0.348784348
	61	−2.328939676	−2.952549358	0.623609682
5-young, 6-old	193	−2.790174327	−4.357471214	1.567296887
	196	−2.399611954	−3.054052171	0.654440217
	198	−2.235703494	−2.938346866	0.702643373
	208	−2.571476271	−3.420631435	0.849155164
	241	−3.367645443	−3.183456559	0.184188884
11-old	246	−3.11809211	−2.06825045	1.049841659

	253	−2.735524232	−2.59539048	0.140133752
	256	−2.994975639	−2.61723673	0.377738909
	769	−2.16887793	−2.725290025	0.556412094
	772	−1.978795615	−2.051087584	0.072291969
	779	−2.319651199	−1.615505797	0.704145402
	784	−2.743607773	−2.584009882	0.159597891
	817	−2.167306264	−2.863109012	0.695802748
	832	−2.588064359	−3.092448819	0.50438446
14-young	961	−2.696765372	−4.257705675	1.560940303
	971	−2.76714535	−3.039556732	0.272411383
	976	−2.868121352	−3.215679271	0.347557919
	1009	−3.921401796	−4.539954538	0.618552742
	1014	−3.424244768	−3.316705833	0.107538935
	1019	−3.532892526	−3.388507306	0.14438522
	1021	−3.382845664	−3.086761634	0.29608403
	1024	−3.938786029	−3.868548084	0.070237944
	3073	−3.924913268	−3.85295172	0.071961548
	3078	−3.510472883	−3.465869397	0.044603486
	3083	−3.500305401	−3.289997329	0.210308071
	3088	−3.988915546	−4.60620449	0.617288944
	3121	−2.822706384	−3.152444679	0.329738294
	3126	−2.713183499	−3.069280595	0.356097095
21-young, 31-old	3136	−2.732736915	−4.276317398	1.543580483
	3265	−2.603500963	−3.091671384	0.48817042
	3280	−2.264129378	−2.944177892	0.680048514
	3313	−2.72750217	−2.535594218	0.191907952
	3325	−2.037186708	−2.032645995	0.004540713
	3328	−2.234158838	−2.758720676	0.524561839
	3841	−2.891083311	−2.525270832	0.365812479
	3844	−2.638521717	−2.555296821	0.083224896
36-old	3851	−3.104133174	−1.965172413	1.138960761
	3856	−3.345139624	−3.173336976	0.171802648
	3889	−2.436041735	−3.281027309	0.844985574
	3899	−2.190202351	−2.7876306	0.59742825
	3901	−2.338674114	−2.94442212	0.605748006
29-young, 41-old	3904	−2.736126301	−4.299713403	1.563587102
	4036	−2.261246526	−2.927274628	0.666028102
	4048	−2.46848167	−2.513844923	0.045363253
	4081	−3.188965735	−3.666711394	0.477745658
	4096	−3.085676438	−3.784651226	0.698974788

Table S8. Energy values of all the states for young and old subjects' average results for ATN network.

Local Minima Index	State Number	Old Energy	Young Energy	Difference
1-young and old	1	−2.451362883	−3.763143104	1.31178022
	4	−2.300168242	−2.674242027	0.37407378
	6	−2.118432119	−2.431588876	0.31315676
	16	−2.23943671	−1.94520401	0.2942327
	49	−2.325252396	−2.932043562	0.60679117
	86	−2.043477691	−2.67006866	0.62659097
	163	−2.100000966	−2.62780591	0.52780494
	171	−2.218017004	−2.606968882	0.38895188
	208	−2.300473784	−2.999128698	0.69865491
	244	−2.396539925	−2.963292461	0.56675254
	251	−2.527070383	−2.385437792	0.14163259
	256	−3.425800449	−4.109677589	0.68387714
	769	−3.432326445	−4.052304328	0.61997788
	774	−2.541059812	−2.482132702	0.05892711
	781	−2.474129305	−3.018950837	0.54482153
	817	−2.312355983	−2.955007071	0.64265109
	854	−2.248979771	−2.495657685	0.24667791
	862	−2.108003782	−2.519353069	0.41134929
	939	−2.054623541	−2.661530008	0.60690647
	976	−2.355478814	−2.85631534	0.50083653
	1009	−2.163537926	−1.647542648	0.51599528
	1019	−2.146551308	−2.215044116	0.06849281
	1021	−2.295332842	−2.489612301	0.19427946
20-young, 14-old	1024	−2.486945504	−3.700666516	1.21372101

Table S9. Energy values of all the states for young and old subjects' average results for SMN network.

Local Minima Index	State Number	Old Energy	Young Energy	Difference
	1	−3.741577495	−3.974538	0.23296051
	47	−0.798991023	−0.389736776	0.40925425
	52	−2.208836642	−2.65762561	0.44878897
	205	−2.288044887	−2.614452687	0.3264078
	210	−0.732325441	−0.50269215	0.22963329
	241	−0.557014757	−1.22868199	0.67166723
	256	−3.648774403	−3.887840438	0.23296051

Table S10. Energy values of all the states for young and old subjects' average results for VIS network.

Local Minima Index	State Number	Old Energy	Young Energy	Difference
	1	−3.026464067	−3.22548168	−0.1990176
2-Young, Old	16	−2.243041022	−2.736298362	−0.4932573
3-Young, Old	113	−2.228295428	−2.652348968	−0.4240535

	128	−2.988567293	−3.175640027	−0.1870727

Table S11. Energy values of all the states for young and old subjects' average results for VIS network.

Local Minima Index	State Number	OLD Energy	Young Energy	Difference
	1	−4.080193848	−4.214186631	−0.1339928
2-young, old	13	−2.872292112	−3.130718308	−0.2584262
	16	−1.606446106	−1.730435616	−0.1239895
	241	−1.568097533	−1.726350739	−0.1582532
	244	−2.88106275	−3.113842714	−0.23278
	256	−4.088237055	−4.127224708	−0.0389877

T-test results:

Two-sample t-test was performed on the potential connectivity signature states and it is observed that some of the networks of FPN, SAN, and ATN satisfy the Bonferroni correction with least p-values. The t-test results for these networks are shown in **Figures 4(a), 5(a),** and **6(a),** respectively, of the main text. The t-test results of the remaining RSNs, which is DMN, SMN, VIS, and AUD networks, are shown in **Figures S5, S6, S7,** and **S8,** respectively.

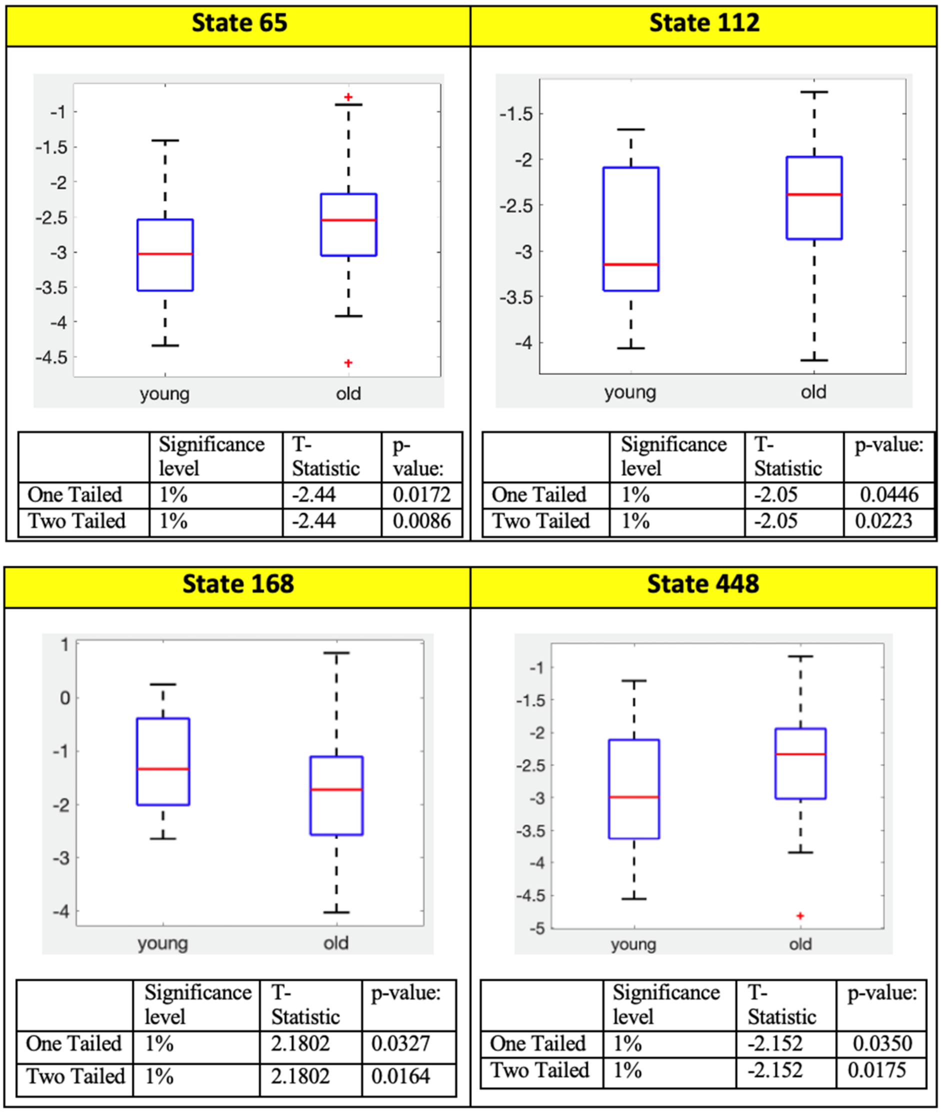

Figure S5. Two-sample t-test was performed on the local minimum states of the DMN whose energy difference was maximum and are considered potential connectivity signatures. The figure shows the two-sample t-test results of the connectivity signature states from **Table S5.**

State 241

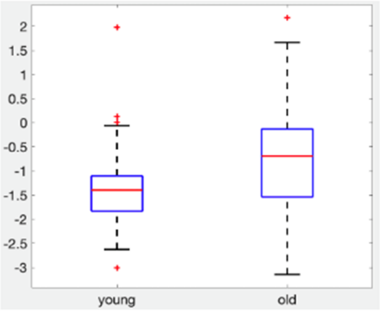

	Significance level	T-Statistic	p-value:
One Tailed	1%	-2.0231	0.0470
Two Tailed	1%	-2.0231	0.0235

Figure S6. Two-sample t-test was performed on the local minimum states of the SMN whose energy difference was maximum and are considered potential connectivity signatures. The figure shows the two-sample t-test results of the connectivity signature states from **Table S9.**

State 16

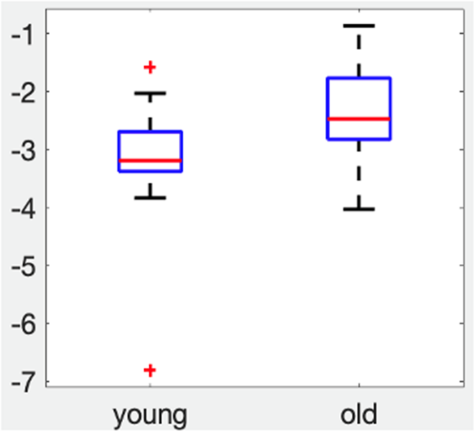

	Significance level	T-Statistic	p-value:
One Tailed	1%	-2.87	0.0063
Two Tailed	1%	-2.87	0.0031

State 113

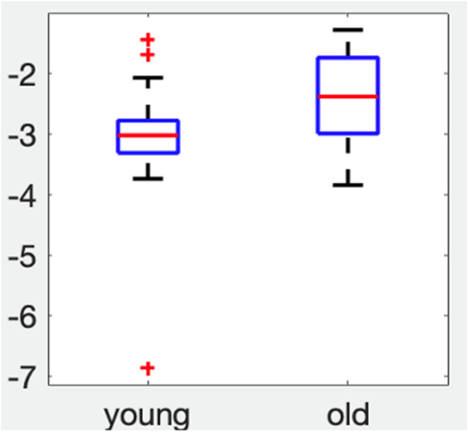

	Significance level	T-Statistic	p-value:
One Tailed	1%	-2.50	0.0162
Two Tailed	1%	-2.50	0.0081

Figure S7. Two-sample t-test was performed on the local minimum states of the SMN whose energy difference was maximum and are considered potential connectivity signatures. The figure shows the two-sample t-test results of the connectivity signature states from **Table S10.**

State 13

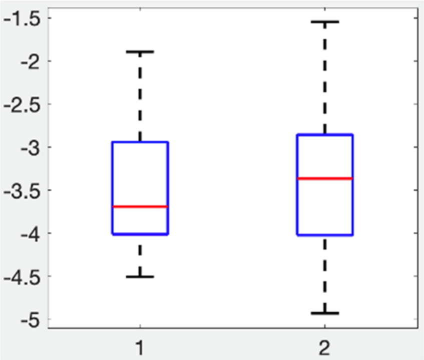

	Significance level	T-Statistic	p-value:
One Tailed	1%	-0.58	0.566
Two Tailed	1%	-0.58	0.2831

Figure S8. Two-sample t-test was performed on the local minimum states of the SMN whose energy difference was maximum and are considered potential connectivity signatures. The figure shows the two-sample t-test results of the connectivity signature state from **Table S11.**

Index	Regions	Subnetwork affiliation
1,2	Precentral gyrus	Sensorimotor
3,4	Superior frontal gyrus, dorsolateral	Frontoparietal
5,6	Superior frontal gyrus, orbital part	Frontoparietal
7,8	Middle frontal gyrus	Salience/frontoparietal/attention
9, 10	Middle frontal gyrus, orbital part	Frontoparietal
11,12	Inferior frontal gyrus, opercular part	Cingulo-opercular
13,14	Inferior frontal gyrus, triangular part	Salience/frontoparietal/attention
15,16	Inferior frontal gyrus, orbital part	None
17,18	Rolandic operculum	Auditory/cingulo-opercular
19,20	Supplementary motor area	Sensorimotor
21,22	Olfactory cortex	None
23,24	Superior frontal gyrus, medial	Default-mode
25,26	Superior frontal gyrus, medial orbital	Default-mode
27,28	Gyrus rectus	None
29,30	Insula	Salience/ cingulo-opercular
31,32	Anterior cingulate and paracingulate gyri	Default-mode/ salience
33,34	Median cingulate and paracingulate gyri	Salience/ cingulo-opercular
35,36	Posterior cingulate gyrus	Default-mode
37,38	Hippocampus	None
39,40	Parahippocampal gyrus	Default-mode
41,42	Amygdala	None
43,44	Calcarine fissure and surrounding cortex	Visual
45,46	Cuneus	Visual
47,48	Lingual gyrus	Visual
49,50	Superior occipital gyrus	Visual
51,52	Middle occipital gyrus	Visual
53,54	Inferior occipital gyrus	Visual
55,56	Fusiform gyrus	Visual
57,58	Postcentral gyrus	Sensorimotor
59,60	Superior parietal gyrus	Salience/attention
61,62	Inferior parietal, but supramarginal and angular gyri	Frontoparietal/attention
63,64	Supramarginal gyrus	Auditory/ cingulo-opercular
65,66	Angular gyrus	Default-mode
67,68	Precuneus	Default-mode
69,70	Paracentral lobule	Sensorimotor
71,72	Caudate nucleus	Subcortical
73,74	Lenticular nucleus, putamen	Subcortical
75,76	Lenticular nucleus, pallidum	Subcortical
77,78	Thalamus	Subcortical
79,80	Heschl gyrus	Auditory
81,82	Superior temporal gyrus	Auditory/attention
83,84	Temporal pole: superior temporal gyrus	Cingulo-opercular
85,86	Middle temporal gyrus	Default-mode
87,88	Temporal pole: middle temporal gyrus	Default-mode
89,90	Inferior temporal gyrus	None

Note that some ROIs were assigned into more than one subnetwork. Odd and even numbers represent left and right hemispheres, respectively. ROI, regions of interest; AAL, Automated Anatomical Labeling.

Figure S9. List of the 90 ROIs defined by the AAL atlas and their subnetwork affiliation (Long, Yicheng et al., 2019).
